# Recognition and management of auditory verbal hallucinations in borderline personality disorder

**DOI:** 10.3389/fpsyt.2025.1511280

**Published:** 2025-02-17

**Authors:** M. B. A. Niemantsverdriet, C. W. Slotema, I. H. A. Franken, J. Brandsma, M. J. P. M. Verbraak, M. L. Deen, J. D. Blom, M. Van der Gaag

**Affiliations:** ^1^ Department of Personality Disorders, Parnassia Psychiatric Institute, The Hague, Netherlands; ^2^ Department of Personality Disorders, Pro Persona, Tiel, Netherlands; ^3^ Department of Psychology, Education & Child Studies, Erasmus University Rotterdam, Rotterdam, Netherlands; ^4^ Faculty of Social and Behavioural Sciences, Leiden University, Leiden, Netherlands; ^5^ Department of Psychiatry, University of Groningen, Groningen, Netherlands; ^6^ Department of Clinical Psychology and Amsterdam Public Health Research Institute, Vrije Universiteit (VU) University, Amsterdam, Netherlands

**Keywords:** antipsychotics, cognitive behavior therapy, psychosis, trauma, voice hearing

## Abstract

**Background:**

Auditory verbal hallucinations (AVH) are experienced by 25% of all people diagnosed with a borderline personality disorder. Since the impact of these hallucinations is often substantial, we asked ourselves how often they are discussed in clinical practice, whether they are treated, and if so, how and to what effect.

**Methods:**

We studied the clinical files of 130 people under treatment at a specialized outpatient clinic for people with personality disorders, and compared the data on the presence and treatment of AVH to those collected in four prior scientific studies by our group.

**Results:**

Of the 130 participants to those earlier studies, 42 had reported on experiencing AVH ≥ once per week. In 31% of the cases this had been missed by the treating healthcare professionals. Of the people suffering from severe AVH, a concurrent schizophrenia spectrum disorder was diagnosed in only 10%. A minority of 41% had received targeted treatment for their AVH. Doses of antipsychotics had been relatively low compared to those recommended for psychotic disorders, and yet all patients treated had reported a decrease in the frequency or severity (or both) of their AVH. Unfortunately, 93% also reported side effects, which led 57% of the group to discontinue this type of treatment. Nonpharmacological treatments such as cognitive behavioral therapy had been offered only to a minority of the participants.

**Conclusion:**

Even in specialized clinical settings it remains challenging to establish the possible presence of AVH in people with a borderline personality disorder. And yet this is worth the effort because of the impact that these hallucinations tend to have, and the positive effects of antipsychotic treatment here presented. Further research is needed to develop algorithms to optimize doses in individual patients, to develop treatment guidelines, and to explore the effects of nonpharmacological treatments.

## 1 Introduction

With a point prevalence of 25%, auditory verbal hallucinations (AVH) are the most prevalent type of hallucination in people with a borderline personality disorder (1, 2). Of these, 40% are either recurrent or persistent in nature (3), which explains at least part of the ensuing burden. However, also less frequent types have the potential to affect people’s lives profoundly (4, 5). This is sadly demonstrated by the established increase in the number of hospitalizations and suicide attempts when compared to other people with borderline personality disorder (6). Curiously, it has long been thought that hallucinations in this group are less severe (7, 8) or that they do not even qualify as hallucinations proper (9), i.e., perceptual experiences which occur in the absence of a corresponding external stimulus and which resemble a veridical perception. The reasoning behind this assumption is illustrated by a qualitative study that explored the response of mental health professionals to reports of psychotic symptoms in patients with borderline personality disorder (10). According to that study, symptoms would sometimes strike the health professionals as so fantastic and bizarre that they doubted the authenticity of the experiences described. Since some patients also showed insight into their experiences, the question arose whether the hallucinations reported on were genuine in nature. Perhaps as a consequence of experiences such as these, hallucinations have never been included in the diagnostic criteria for borderline personality disorder such as those issued by the American Psychiatric Association (11) or singled out as specific targets for treatment in international treatment guidelines (e.g. 12). Of note, the latter does offer recommendations for the treatment of psychotic symptoms in this group, but none of them specifically tailored to AVH. It isn’t hard to see that this omission works both ways: AVH in borderline personality disorder were never included in manuals such as these because they used to be underrated, and they are still frequently underrated because they do not feature in the manuals. And yet phenomenologically, AVH in borderline personality disorder do not differ from those documented in people diagnosed with a schizophrenia spectrum disorder (9, 13–16), while scores for ensuing distress are even higher (14). On average, AVH in borderline personality disorder are less prevalent than those in patients with schizophrenia spectrum disorders, although the frequency of hallucinations and the mean duration (i.e. 17 years for AVH in borderline personality disorder) are quite similar (5, 13, 14). AVH in borderline personality disorder are often present in an intermittent or persistent pattern. Especially the persistent ones can be severe, and cause disruption to people’s lives (3). As to increases in the severity and frequency of hallucinations in relations to stress, this is something that both patients with borderline personality disorder and those with schizophrenia spectrum disorder although patients the first group display the strongest reactivity (17). Other similarities can be found in the neurobiological mechanisms underlying AVH. In borderline personality disorder, brain activation during AVH as well as functional connectivity resemble those in schizophrenia spectrum disorders (18, 19). The same holds true for auditory sensory gating deficiencies although to a lesser extent (20).

Moreover, even the mediating role of childhood trauma has been established as significant for both groups (2, 21). These findings underscore the need to take AVH in borderline personality disorder as seriously as we do in people with psychotic disorders, and to develop evidence-based strategies for their treatment.

### 1.1 Study aims

To that end, an obvious avenue to explore would be the use of antipsychotics. However, studies in this area are few and moreover do not explicitly report on the effects of these medicines on AVH. Instead, they usually render the effects of antipsychotics upon positive symptoms in general (in these cases comprising chiefly hallucinations, delusions and dissociation) and sometimes also on behavioral symptoms such as hostility and aggression. Meta-analyses indicate that antipsychotics have a small but significant effect upon psychoses in this group, with a standardized mean difference of 0.23 for cognitive and perceptual symptoms (22, 23). A systematic review by our group summarized the results of 21 pharmacological studies, only four of which had exclusively focused on people with borderline personality disorder *and* psychotic symptoms (1). Despite this modest number, we nonetheless concluded that both first- and second-generation antipsychotics appear to have positive effects on the severity of psychotic symptoms in this group. In an attempt to refine these results, the present study seeks to establish to what extent AVH are recognized in people with borderline personality disorder in clinical practice, and how they are being managed. With these goals in mind, our study aims to answer the following questions:

1. What is the point prevalence of AVH in people with borderline personality disorder at a tertiary outpatient clinic for people with personality disorders?
2. How many of these people were classified with a comorbid diagnosis of schizophrenia spectrum disorder?
3. Do these people receive treatment for their AVH, and if so, what type of treatment?
4. If antipsychotics are being prescribed, are dosages consistent with prevailing prescription guidelines for psychotic symptoms?
5. What are the effects and side effects of these medicines in people with borderline personality disorder?

## 2 Methods

We conducted a retrospective chart review at a tertiary outpatient clinic for personality disorders at Parnassia Psychiatric Institute in The Hague which offers state-of-the-art evidence-based treatments. People diagnosed with a borderline personality disorder according to DSM-IV or -5 criteria (24, 25) were selected from four previous studies carried out by our research group (published on in 2, 26, 27). These studies had been approved by the Dutch National Medical Ethical Committee (NL1371209706) on 09-22-2006, and by the Committee for Medical Ethics of Leiden University Medical Center on 08-10-2018 (NL64839.058.18) and 03-14-2019 (NL66211.058.18). Written informed consent was obtained from all participants.

Of these above mentioned people we selected those who had either reported experiencing AVH at least once a week, or had never experienced such hallucinations. In the group with AVH the frequency of these hallucinations and the intensity of the ensuing distress and disruption to life had been assessed using the *Psychotic Symptom Rating Scale, AVH-Related Items* (PSYRATS-AHRS; 28) and the *Questionnaire for Psychotic Experiences* (QPE; 29). Severe distress was operationalized as a score of ≥ 3 on the PSYRATS-AHRS item ‘Intensity of distress’ and the QPE item ‘Experienced distress’. Significant disruption to life was operationalized as a score of ≥ 2 on the PSYRATS-AHRS item ‘Disruption to life’ and the QPE item ‘Impact’. From the files we extracted people’s age, gender, comorbid DSM-IV or DSM-5 diagnoses, and prescribed psychotropic medication from the previous studies’ baseline data (2, 26, 27). We then explored the original case notes from 12 months before up to 12 months after study entry to obtain information on the presence of AVH during psychiatric consultations, reasons for not attributing these hallucinations to another DSM-IV or DSM-5 disorder, treatments given to target these AVH, indications for antipsychotic prescription, maximum doses of antipsychotics, and self-reported effects and side effects of these medicines. To obtain olanzapine-equivalent doses, we used the conversion method from Gardner et al. (30) international consensus study. The olanzapine-equivalent dose for pipamperone was derived from Leucht et al. (31) ‘defined daily doses’ (DDD) method. The data were extracted from the chart files by a psychiatrist and resident in psychiatry.

### 2.1 Statistics

To compare the groups with and without AVH the following analyses were used. Because of a non-normal distribution, Mann-Whitney testing was used to compare the means used for age, number of comorbid diagnoses, and number of psychotropic medications, while chi-square analyses were used for frequencies of gender and types of psychotropic medication, and Fisher’s exact test in case of small (expected) frequencies. The relationship between antipsychotic prescription and the reported presence of AVH was also tested with chi-square analyses. Using Mann-Whitney tests we compared maximum antipsychotic doses for the participants with and without AVH and for two groups of indications for prescribing them (i.e. antipsychotics for AVH or other positive symptoms of psychosis vs. non-psychotic symptoms). Antipsychotic doses were reported in terms of medians in case of non-normal distribution. For all analyses, p-values below.05 were considered statistically significant; in cases of multiple comparisons, Benjamini and Hochberg (32) false discovery rate correction was applied.

## 3 Results

**Figure 1:**
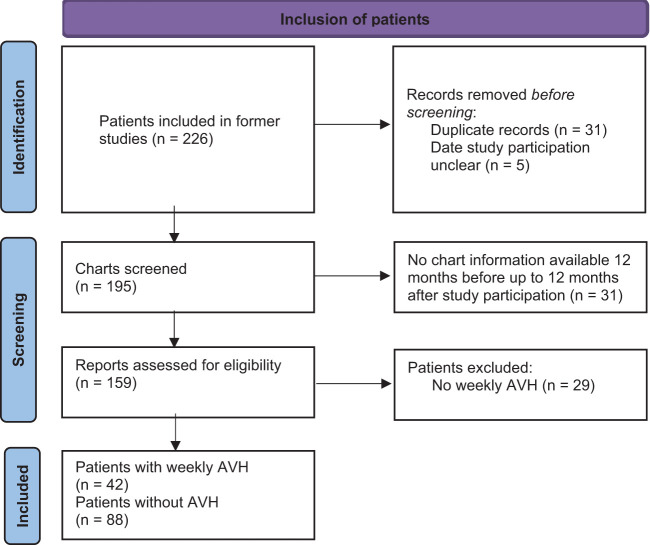
PRISMA flow diagram.

From the 226 people who participated into prior studies we selected those who had received treatment for borderline personality disorder at the outpatient clinic within 12 months before and after entering the original study in which they had participated (n=195). We then removed 31 duplicate files of people who had participated more than once in these studies, and five files pertaining to people with unclear dates of participation. Among the remaining 159 people, 42 had been hearing voices at least once a week, and 88 had never or only rarely experienced them (see **Figure 1** for a PRISMA flow diagram).

### 3.1 Demographics

**Table 1** shows the demographic characteristics of the participants with and without AVH, as well as the medication they had used derived from the four previous studies. The groups did not differ significantly regarding these variables, except for the use of antipsychotics. Of the 42 participants who had reported on experiencing AVH in the previous studies, 31% had no references to these hallucinations in their clinicians’ charts.

**Table 1:** Demographic characteristics of and medication use by participants with and without auditory verbal hallucinations (N=130).

	Participants with AVH (n=42)	Participants without AVH (n=88)	Z- or χ^2^ value	*p* ^a^
Age in years, *median* (range)	31.5 (19-58)	34 (21-67)	Z = -1.08	.84
Female, n (%)	40 (95%)	80 (91%)	χ^2^ = 0.75	.90 ^b^
Comorbid diagnoses, n (range)	1.5 (0-4)	2 (0-5)	Z = -0.42	.90
Psychotropic medications, *median* (range)**	2 (0-6)	1 (0-5)	Z = -1.97	.22
Antipsychotics, n (%)	17 (46%)	17 (20%)	χ^2^ = 8.63	0.03^b^
Mood stabilizers, n (%)	3 (8%)	8 (9%)	χ^2^ = 0.05	1.00^b^
Antidepressants, n (%)	16 (43%)	40 (47%)	χ^2^ = 0.15	.90
Anxiolytics, n (%)	13 (35%)	25 (29%)	χ^2^ = 0.39	.90
Stimulants, n (%)	3 (8%)	6 (7%)	χ^2^ = 0.042	1.00^b^
Other, n (%)	6 (16%)	2 (2%)	χ^2^ = 8.09	.09^b^

^a^ Benjamini-Hochberg corrected p-values; ^b^ Fisher’s Exact Test; ** Other than antipsychotics.

### 3.2 Diagnoses in the schizophrenia spectrum

In the group of people with AVH, the majority (69%) had reported that these hallucinations had caused severe distress and/or disruption to life. Of them, only 10% had been diagnosed with a schizophrenia spectrum disorder (i.e. brief psychotic disorder or psychotic disorder not otherwise specified in accordance with DSM-IV criteria, or unspecified schizophrenia spectrum or other psychotic disorder in accordance with DSM-5 criteria). None of the other participants had been diagnosed with schizophrenia or schizoaffective disorder. **Table 2** presents possible reasons for not classifying AVHs according to DSM-criteria.

**Table 2:** Reasons for not classifying AVHs according to DSM-criteria.

Reason	n (%)
AVH were not discussed	8 (31%)
No reason reported in medical file	10 (39%)
Consequence of traumatic experiences	2 (8%)
Diagnostic process not finished	2 (8%)
Attributed to borderline personality disorder	1 (4%)
Attributed to mood disorder, PTSD, dissociation and personality disorder	1 (4%)
Attributed to mood disorder, PTSD, anxiety disorder and personality disorder	1 (4%)
Patient reported no distress from AVH	1 (4%)

AVH, auditory verbal hallucinations; PTSD, posttraumatic stress disorder.

### 3.3 Treatments and treatment outcomes

Of the whole group of 130 participants to our study, 39% had been using antipsychotics. Stated indications for this comprised psychotic symptoms (33%), sleep disturbances (31%), affective dysregulation (8%), impulse dyscontrol (8%), anxiety (6%), and other symptoms (14%). The 42 participants who had reported on experiencing AVH had been using antipsychotics more often than those who had not (62% vs. 28%, p<.001). However, only 41% of the people who had experienced severe distress or disruption to life had been receiving targeted treatment (see **Table 3**). Three of them (12%) had been offered non-pharmacological treatments for their AVH, i.e. cognitive behavioral therapy for psychosis (CBTp) or a smartphone application offering language games to reduce AVH-associated distress and improve social functioning (Temstem; 33, 34). One participant had received both antipsychotics and CBTp. Of note, in only 54% of the cases the antipsychotic medications prescribed to people with AVH had been prescribed with the *specific aim* of treating these hallucinations.

**Table 3:** Treatment of AVH in patients with BPD.

Treatment	Participants with severe AVH (n=29)	Participants with mild AVH (n=13)
Antipsychotics, n (%)	10 (34%)	2 (15%)
CBTp, n (%)	2 (7%)	0
Temstem, n (%)	1 (3%)	0

Severe AVH = AVH causing severe distress and/or disruption of life, CBTp = cognitive behavioral therapy for psychosis.

The people with AVH had received significantly higher doses of antipsychotics than those without (with AVH: median 7.11 mg; without AVH: median 1.35 mg; *z*=-3.03, *p*=0.002). For the 14 people taking antipsychotics for their AVH, the median maximum olanzapine-equivalent dose had been 5.86 mg (SD 5.99). In 12 people the effectiveness of treatment had been evaluated by the clinician, with all 12 (100%) reporting a decrease in the occurrence of AVH or in AVH-associated distress or both. Of them, 93% reported side effects such as sedation (64%), extrapyramidal symptoms (64%), and metabolic syndrome (21%). Other side effects (all reported only once) were a depressed mood, sleep disturbances, memory loss, visual hallucinations, nasal congestion, salivation, QT prolongation, galactorrhea, elevated liver enzymes, itching, urinary incontinence, and constipation. Of the antipsychotics prescribed for AVH, 57% had been discontinued because of the side effects. For three antipsychotic treatments (10%) the reason for discontinuation had not been mentioned. An analysis of the eight people who had received antipsychotics for the treatment of psychotic symptoms in general (comprising AVH, other hallucinations, and delusions) revealed a mean maximum olanzapine-equivalent dose of 4.95 mg (SD 3.12). Of the 11 antipsychotic treatments given in this group, 45% had been discontinued due to stated side effects; for two treatments no reason for discontinuation had been given. All eight participants had reported a decrease in AVH frequency or AVH-associated distress or both.

## 4 Discussion

In this retrospective chart study we evaluated the recognition and management of auditory verbal hallucinations (AVH) in people diagnosed with a borderline personality disorder under care at a specialized outpatient clinic. Judging by the medical files that we studied, in almost a third of the cases this symptom had been missed by the treating healthcare professionals, while less than half of the people with severe AVH had been offered targeted treatment. In those cases where treatment had been offered, antipsychotics had been the first choice. This invariably resulted in a decrease of the occurrence of AVH, a decrease in AVH-associated distress, or both. A drawback was that 93% of the people treated went on to experience side effects, which prompted 57% to discontinue the use of these medicines. Non-pharmacological treatments had been offered only to a small minority of the voice hearers.

These findings indicate that even in specialized psychiatric settings it is not always easy for people with borderline personality disorder to discuss their AVH. Possible contributing factors are the reluctance that some people may have to disclose the presence of AVH (e.g. out of shame or out of fear of being diagnosed as psychotic; 35, 36), the massive salience of other problems at hand (e.g. interpersonal conflicts, financial problems, suicidality), and their own ignorance of the potential effects of AVH on their well-being. Especially when health professionals are insufficiently aware of these mechanisms, or worse, may not realize that AVH can be experienced in this patient group, this may lead to under recognition and hence also undertreatment. Studies show that this, in turn, may result in more severe distress and dysfunctioning in a group of people who already tend to have much to cope with in life (5, 10, 36).

And yet, even when both patient and health professional fully realize that these phenomena deserve attention, the diagnostic work-up may remain quite challenging. Internationally accepted diagnostic criteria such as those featuring in the DSM-5 necessarily lag somewhat behind scientific developments. The idea that we may not do justice to AVH in people with borderline personality disorder by calling them ‘pseudohallucinations’ or by characterizing them as ‘brief and transient’ when they are not, is relatively recent. Since AVH are not diagnostic or even adjuvant criteria in manuals such as the DSM, healthcare professionals may go on to dismiss these symptoms as somehow ‘unreal’ or associate them primarily with schizophrenia spectrum disorders. One of the more remarkable findings in our study was that only 10% of the participants with severe AVH was diagnosed with a comorbid schizophrenia spectrum disorder. This indicates that it is worthwhile to keep an open mind when dealing with people with borderline personality disorder who claim to be hearing voices, and to accept that our clinical realities may not always tally with what is stated in textbooks and diagnostic guidelines.

### 4.1 Treatment with antipsychotics

Regarding treatment, our results indicate that antipsychotics appear to be effective for voices in this group. This holds true for both first- and second-generation antipsychotics. Side effects were reported on by 93% of the participants, which is quite similar to the rate found in females diagnosed with a schizophrenia spectrum disorder (approximately 80%, 37). Likewise, the proportion of people who discontinued the use of antipsychotics (57%) is in line with the findings for females with a schizophrenia spectrum disorder (53%, 38). The number of reported side effects suggests that further research is needed to establish which antipsychotics yield optimal results in which dosages and under which circumstances. As we saw, the median maximum olanzapine-equivalent dose of in the present study was 5.86 mg. In the absence of international guidelines for the specific treatment of AVH in borderline personality disorder we compared this to doses in schizophrenia spectrum disorders. In these disorders, antipsychotics and CBTp are the most widely applied treatments (39, 40), with recommended daily doses of 10-20 mg olanzapine or equivalents thereof (30), and a minimum effective dose of 7.5-10 mg (41, 42). In the Dutch Multidisciplinary Clinical Guideline for Personality Disorders, recommended olanzapine-equivalent doses lie between 2.5 and 10 mg (43). It should be noted however, that these doses are derived from studies on ‘cognitive-perceptual symptoms’ (a term used to cover delusions, hallucinations, and instances of dissociation), mood dysregulation, and impulsivity (44–49). The Dutch guideline moreover does not specify optimal effective doses. As a consequence, it is unclear how these recommended doses relate to the median maximum dose of 5.86 mg found in the present study. Taken at face value, this median does lie squarely within the more prudent ranges for people with schizophrenia spectrum disorder. And yet the high rate of side effects suggests that, on average, the doses given may have been too high. A possible reason for this high rate may have been that almost all participants to our study were female, and that females with a schizophrenia spectrum disorder who undergo antipsychotic treatment are reported to have significantly higher dose-adjusted concentrations for the majority of antipsychotics, thereby increasing the risk of side effects (50). The same may be true for our patients with borderline personality disorder. In clinical practice, we therefore recommend starting with a low dosage of antipsychotics and gradually increasing the dosage in small increments, while carefully monitoring both the beneficial and adverse effects of these medications for each individual patient. Whether that will be successful is in need of further study though.

### 4.2 Nonpharmacological treatments

As we saw, a minority of the people studied here had been treated with CBTp (to decrease the distress caused by AVH by challenging thoughts, beliefs, attitudes, and their associated behaviors), and Temstem, a smartphone application to reduce AVH-related distress either in combination with antipsychotics or as monotherapies. So far these techniques do not feature in consensus guidelines for their efficacy in the treatment of AVH in people with borderline personality disorder. Nonetheless, the Dutch *Hearing Voices* guideline recommends CBTp for people suffering from AVH, if only for the reason that cumulative meta-analyses indicate a positive effect of it upon hallucinations and delusions, irrespective of a person’s clinical diagnosis (51, 52). The guideline goes on to recommend antipsychotics for people who do not benefit sufficiently from CBTp (51). Another promising technique is repetitive transcranial magnetic stimulation (rTMS). Several studies found positive effects of rTMS on (medication-resistant) AVH, although mainly in schizophrenia spectrum disorders (53, 54). Yet another technique that might turn out to be effective is trauma-focused therapy with the aid of eye movement desensitization and reprocessing (EMDR). Its efficacy was investigated in people with borderline personality disorder and comorbid PTSD, with a subgroup of people (n = 11) experiencing AVH. For the whole study sample (N=47) no statistically significant effects were found, but the AVH-subgroup did show positive results (unpublished results, 27). Moreover, studies on the effectiveness of trauma-focused therapies to ameliorate psychotic symptoms in general, which are currently accumulating, show promising results (50). Although the latter studies mainly included people with schizophrenia spectrum disorders and comorbid posttraumatic stress disorder (PTSD), we believe that people with borderline personality disorder and especially those with AVH may also benefit from these treatments since the prevalence of PTSD and (childhood) trauma is high in this group, too (i.e., 63% for PTSD in the group with hallucinations (2)). A final technique that deserves to be mentioned is psychotherapy, the preferred type of treatment for people with personality disorders in general (55, 56). Dialectical behavior therapy, one of the most widely provided psychotherapies for borderline personality disorder, aims to help people regulate the intensity of their emotions (57). Since stress-induced emotions have been found to be associated with the severity of hallucinations (58, 59), an improvement in emotion regulation may theoretically help to reduce the severity of AVH in this group (60, 61). Thus, in addition to treatment with antipsychotics, there would seem to be several nonpharmacological treatments that may prove to be effective in reducing the frequency and burden of AVH in borderline personality disorder. Given the high prevalence and often debilitating effects of AVH in this group, there is an urgent need for clinical trials investigating this.

### 4.3 Limitations

An important limitation to the present study is the relatively small sample size of people with AVH (n=42). A second limitation is the retrospective nature of our study, and a third that it was carried out at a single secondary outpatient clinic. Fourth and finally, by focusing on an observational time frame of two years, we were unable to gain a complete overview of the participants’ medical history. Crucially, we were unable to retrieve data on the full duration of antipsychotic treatment and on the effects and side effects during each phase, for the reason that a large proportion of the participants had started or ended their treatment outside the study period. For example, if an antipsychotic had been started before the study period, initial, transient side effects may have been missed. In that sense some caution is warranted in interpreting our results.

## 5 Conclusions

In our retrospective chart review of 130 people treated for borderline personality disorder at a specialized outpatient clinic we found that the presence of AVH had remained undiscussed in 31% of the cases. We moreover found that not all the people recognized as voice hearers had been offered targeted treatment for their AVH, and in the group of those who suffered severely even less than half. Most of those who had been offered treatment had been given antipsychotics, which were effective or partly effective, but in 93% of the cases yielded side effects even though dosages had been rather low compared to dosages recommended for the treatment of positive symptoms of psychosis in schizophrenia spectrum disorders. More than half therefore discontinued the use of antipsychotics, citing said side effects as the main reason for doing so. Nonpharmacological treatments such as CBTp and Temstem had been offered only to a small minority of the voice hearers. Neither these techniques nor antipsychotic treatments have been registered as evidence-based treatments for AVH in borderline personality disorder. Our findings therefore imply that all these treatments are in need of further study for this specific indication in this specific group, and that there is an urgent need to do so because of the relatively high burden and severe disruption to life associated with voice hearing. On the basis of our findings our practice-based advice is to i) systematically assess the presence of AVH in people with borderline personality disorder, ii) when needed, initiate antipsychotic treatment at relatively low dosages and then slowly titrate upwards, and iii) carefully monitor effects and side effects in each individual patient to establish their personal optimal dose. As to future scientific research, our study indicates that we are in need of (randomized) controlled trials to investigate the efficacy of the various pharmacological and nonpharmacological interventions currently available, and on the basis of these findings develop evidence-based treatment protocols.

## Data Availability

The original contributions presented in the study are included in the article/supplementary material. Further inquiries can be directed to the corresponding author/s.
